# Microbial diversity and community assembly in heavy metal-contaminated soils: insights from selenium-impacted mining areas

**DOI:** 10.3389/fmicb.2025.1561678

**Published:** 2025-04-14

**Authors:** Zhiyong Wang, Guangai Deng, Chongyang Hu, Xue Hou, Xinyuan Zhang, Zhiquan Fan, Yong Zhao, Mu Peng

**Affiliations:** ^1^Hubei Key Laboratory of Biological Resources Protection and Utilization, Hubei Minzu University, Enshi, China; ^2^College of Biological and Food Engineering, Hubei Minzu University, Enshi, China; ^3^College of Life Science, Baicheng Normal University, Baicheng, China

**Keywords:** selenium, heavy metal, bacterial diversity, community assembly, stochastic and deterministic processes

## Abstract

The mining industry in China plays a pivotal role in economic development but also leads to severe environmental issues, particularly heavy metal pollution in soils. Heavy metal pollution significantly impacts soil microbial communities due to its persistence and long-term residual effects. We assessed changes in microbial diversity, community structure, and assembly mechanisms in selenium-impacted soils. This study investigates the impacts of selenium (Se) and other heavy metals on soil microbial communities in selenium-rich mining areas using full-length 16S rRNA gene sequencing. Our results showed that Se and other heavy metal contamination significantly altered microbial community composition, favoring metal-tolerant phyla such as Proteobacteria, Actinobacteriota and Firmicutes, while reducing the abundance of sensitive groups like Acidobacteriota and Chloroflexi. Microbial diversity decreased as Se and other heavy metal concentrations increased. Mantel test analysis revealed that soil total potassium (TK), soil organic carbon, total nitrogen, and several other metals, including zinc, niobium, titanium (Ti), manganese, rubidium, barium, potassium, cobalt, gallium (Ga), Se, chromium (Cr), vanadium, and copper were significantly and positively correlated with microbial community composition across all soil samples. Random forest analysis showed that soil TK and multiple elements [Cr, Ti, nickel (Ni), Ga and Se] were the most important predictors of bacterial diversity, emphasizing the role of multiple elements in shaping microbial communities. Co-occurrence network analysis revealed that Se and other heavy metal contamination reduced network complexity and stability, with high Se-contaminated soils exhibiting fragmented microbial networks. Community assembly was primarily driven by drift in control soils, whereas dispersal limitation became more prominent in Se-contaminated soils due to heavy metal toxicity. These findings highlight the ecological consequences of heavy metal pollution on microbial communities and offer valuable insights for effective soil management and remediation strategies.

## 1 Introduction

In China, the mining industry plays a crucial role in socio-economic development, but also causes severe ecological and environmental challenges, particularly heavy metal pollution in soils (Marimuthu et al., 2021; Wen et al., 2024). Among these pollutants, selenium (Se) is of particular concern due to its dual nature—it is an essential micronutrient at trace levels but becomes toxic at higher concentrations, posing significant risks to soil ecosystems, water quality, and agricultural productivity by disrupting microbial functions, soil fertility, and plant growth (Banerjee et al., 2022; Ma et al., 2024). Se contamination in mining regions is exacerbated by the release of Se-rich tailings and wastewater, which can infiltrate adjacent soils, altering their physicochemical properties and harming the microbial communities that are crucial for ecosystem stability (Rosenfeld et al., 2018; Peng et al., 2024). The impact of Se on soil microorganisms is especially critical, as these organisms play essential roles in nutrient cycling and maintaining ecological balance (Yang R. et al., 2022). When exposed to elevated Se levels, soil microbial communities may undergo significant shifts, with certain taxa either adapting to or being suppressed by the contaminant. These changes can have profound effects on microbial biodiversity, community structure, and soil functions such as carbon and nitrogen cycling (Rijk, 2023).

Soil microorganisms, which are primary drivers of nutrient cycling and ecosystem stability, are highly susceptible to heavy metal contamination (Tang et al., 2019). Their high surface-area-to-volume ratio, coupled with their physiological diversity, makes microbial communities both sensitive indicators and key mediators of soil responses to metal stress (Ma et al., 2022; Sarkar et al., 2022). Heavy metal contamination significantly alters microbial biomass, community structure, and diversity, frequently favoring metal-tolerant groups such as Proteobacteria and Firmicutes, while suppressing metal-sensitive taxa, including members of Actinobacteria and Acidobacteria (Zhao et al., 2019; Hemmat-Jou et al., 2021). These shifts disrupt critical ecosystem functions, including carbon and nitrogen cycling, and impair the soil’s capacity to support plant growth (Zhou et al., 2016; Rijk and Ekblad, 2020). Although there is substantial research on the effects of heavy metals like cadmium (Cd) and arsenic (As) on microbial communities (Olaniran et al., 2013), the specific impact of Se contamination and its interaction with microorganisms in mining environments remains incompletely understood. Selenium’s behavior in soil is influenced by various factors, including its chemical form and the presence of other contaminants, making it complex to investigate how microbial communities respond to varying Se contamination levels (Rosenfeld et al., 2018). High-throughput sequencing technologies and metagenomics offer powerful tools to explore these interactions in detail, enabling a deeper understanding of how microbial communities adapt and function under Se and other heavy metal stress (Xiao et al., 2019; Yan et al., 2020).

The assembly processes of microbial communities are key determinants of microbial diversity and composition, especially under heavy metal stress (Chen et al., 2024; Sun et al., 2024). These processes are governed by two principal ecological mechanisms: deterministic processes, driven by environmental filtering and niche selection, and stochastic processes, which include dispersal limitation, mass effects, and random demographics (Caruso et al., 2011; Chase and Myers, 2011; Dini-Andreote et al., 2015). In heavy metal-contaminated soils, deterministic processes often dominate due to the strong selective pressures exerted by metal toxicity and altered soil chemistry, such as reduced pH or elevated ionic strength (Zhang et al., 2022; Sun et al., 2024). These pressures favor microbial taxa with specialized traits, such as efflux pumps and enzymatic detoxification pathways, which provide resistance to metals (Silver, 1996; Sharma et al., 2024). Conversely, in less polluted or more heterogeneous environments, stochastic processes play a more significant role, as random factors influence microbial distribution (Zhang et al., 2022; Liu et al., 2024). Although deterministic processes are generally regarded as the primary drivers in heavily polluted environments, the relative contributions of deterministic and stochastic factors along Se and other heavy metal contamination remain poorly quantified. In addition to assembly mechanisms, microbial interactions within communities are critical to their resilience and functionality in stressed environments (Nizamani et al., 2024). Under heavy metal stress, cooperative interactions, such as nutrient sharing and mutual detoxification, often emerge as survival strategies, leading to the formation of tightly connected microbial networks (Sarsan et al., 2021). Advanced network analysis methods, including those using random matrix theory (RMT)-based molecular ecological network (MEN) analysis, offer robust frameworks for quantifying these interactions and assessing microbial community stability (Deng et al., 2012). Recent studies have shown that the complexity and connectivity of microbial networks are positively correlated with ecosystem resilience, suggesting that such networks play a pivotal role in maintaining functionality under heavy metal stress (Chen et al., 2021; Wu et al., 2024). However, the links between microbial interaction networks and community assembly processes in Se-contaminated soils remain underexplored.

To address these research gaps, this study aims to systematically investigate the effects of Se and other heavy metal pollution on soil microbial communities. Specifically, we focus on: (1) characterizing shifts in microbial community structure and diversity along gradients of Se and other heavy metal contamination; (2) quantifying the relative contributions of deterministic and stochastic processes to microbial community assembly under varying Se and other heavy metal contamination; and (3) elucidating the role of microbial interactions in maintaining community stability and functionality. By integrating high-throughput sequencing and advanced ecological modeling, this study provides comprehensive insights into the interplay between Se, other heavy metal pollution, and soil microbial ecology, contributing to the development of sustainable soil management and remediation practices.

## 2 Materials and methods

### 2.1 Soil collection and treatments

Soil samples were collected from Enshi, Hubei Province, China, which is known as the only Se mine in the world (Zhu and Zheng, 2001). Samples were collected from two different contamination levels (30°10′N, 109°46′E, elevation 1,600 m): a selenium-rich carbonaceous siliceous rock area (HSe), which is heavily contaminated by Se, and a relatively less contaminated area located 100 m away from the selenium-rich site (LSe). A non-contaminated region with similar soil parent material similar to that of the contaminated regions was selected as the control (Control) without Se contamination. Ten independent sampling points were randomly selected within each region (HSe, LSe, Control), with each sample representing an independent biological replicate. The surface soil was collected from the top 0–20 cm layer. At each sampling point, the soil was collected using a clean soil auger, and composite samples were created by mixing soil from five sub-sampling points within a 1-meter radius around each random point. Each composite sample represented one biological replicate. The samples were then divided into two subsamples: one for measuring soil indicators and the other stored at −80°C for subsequent high-throughput sequencing analysis. Soil moisture content was measured before the soil samples were subjected to natural air-drying. Freshly collected soil samples were weighed immediately after collection to determine their moisture content. Soil moisture content was determined using the drying and combustion method.

After natural air-drying, the soil samples were sieved through a 2 mm mesh to homogenize them, removing fine roots and stones. Soil pH was measured using a pH meter with a soil/water ratio of 5 g/25 mL. Soil organic carbon (SOC) and total nitrogen (TN) were determined using an elemental analyzer (Elementar, Hesse, Germany). Total potassium (TK) content was measured using the NaOH fusion-flame photometry method. Available potassium (AK) was extracted with 1 mol/L ammonium acetate and determined by flame photometry. Total phosphorus (TP) was determined using sulfuric acid-perchloric acid digestion followed by atomic spectroscopy. Available phosphorus (AP) was extracted with 0.5 mol/L NaHCO₃ and analyzed using the molybdenum-antimony anti-spectrophotometric method (Olsen method). Alkali-hydrolyzed nitrogen (AN) was determined using the alkali diffusion method. Soil metal elements contents were determined by inductively coupled plasma mass spectrometry (ICP-MS). Statistical analysis was performed using one-way analysis of variance (ANOVA) to determine the significant differences among the different pollution areas, with a significance level set at *p* < 0.05.

### 2.2 DNA extraction, library construction, and sequencing

A total of 30 soil samples were collected, and high-quality genomic DNA was extracted from 0.1 g of soil using a commercially available soil DNA extraction kit (Power Soil DNA Isolation Kit, Qiagen) following the manufacturer’s protocol. The purity, concentration, and integrity of the extracted DNA were assessed using a Nanodrop spectrophotometer (Thermo Fisher Scientific, Waltham, MA, United States), and the fragment size distribution was examined by agarose gel electrophoresis. The full-length 16S rRNA gene was amplified using specific primers (27F 5′-AGRGTTTGATYNTGGCTCAG-3′, 1492R 5′-TASGGHTACCTTGTTASGACTT-3′). The PCR products were precisely quantified using a Qubit fluorometer (Thermo Fisher Scientific, Waltham, MA, United States), and the fragment size distribution of the amplicons was verified by agarose gel electrophoresis. The amplified products then underwent end repair and dA-tailing. Sequencing adapters were ligated to the ends of the repaired products, followed by purification of the constructed libraries using magnetic beads. The final libraries were loaded onto an R10.4 Flow Cell, and sequencing was performed on a PromethION P48 sequencing platform (Oxford Nanopore Technologies, Oxford, United Kingdom).

### 2.3 Data process and statistical analyses

The raw sequencing data were processed using Guppy base-calling software version 5.0.16 (Oxford Nanopore, Oxford, United Kingdom) with default parameters to generate high-quality reads. Adapter and barcode sequences were trimmed using Porechop (version 0.2.3). Quality filtering was performed using NanoFilt (version 2.7.1) to exclude sequences with a quality score below 10, and Cutadapt (version 3.5) was employed to remove length discrepancies and obtain clean, primer-free sequences. Chimeric sequences were identified and removed with Minimap2 (version 2.17) and yacrd (version 0.6.2). Finally, taxonomic assignment of ASVs was performed using QIIME2 (version 2022.3) based on the SILVA 138 release.

Alpha diversity indices, including abundance-based coverage estimators (ACE), Shannon, Chao1, and Simpson, were calculated to evaluate microbial community richness and diversity. Beta diversity metrics, such as Bray–Curtis dissimilarity, were computed to assess the differences of community composition. The relationships between examined soil variables and the dominant bacterial phyla or genus were displayed with a Spearman correlation heatmap, and visualized with the R package ggplot2. Principal component analysis (PCA) was performed to visualize microbial community variations among samples. Additionally, three non-parametric methods—Adonis, ANOSIM, and MRPP—were employed to analyze overall bacterial community structure. Redundancy analysis (RDA) was conducted to investigate the relationships between soil bacterial communities and environmental factors, with significant variables identified via the envfit function in the vegan package using 999 permutations. The Mantel test was employed to evaluate correlations between soil physicochemical properties and bacterial community structure, and the results were visualized with heatmaps generated using the pheatmap package. The relative abundance-weighted community degree (RACD) was calculated to examine the variations of diazotrophic community associations by different components, as follows: ∑*N* × RA, where *N* is the number of ASVs, and RA is the relative abundance of the ASVs (Sun et al., 2020). A random forest model was applied to identify the importance of soil variables in predicting the microbial diversity indices. Predictor importance was assessed based on the percentage increase in mean squared error (MSE), and the significance of each predictor was validated using the R package “randomForest” (R version 4.3.3).

### 2.4 Co-occurrence network analysis and community assembly

A co-occurrence network analysis was conducted using the phylogenetic molecular ecological network analysis pipeline (pMENA) to explore relationships between different microbial taxa (Deng et al., 2012). Amplicon sequencing variants (ASVs) only present in over eight samples were retained for subsequent analysis. Low-abundance taxa were filtered out from the ASV table to reduce interference. Network topological properties, including node degree, betweenness centrality, average clustering coefficient and modularity, were calculated to identify key taxa and network modules. The networks were visualized using Gephi software (Bastian et al., 2009). The vulnerability and robustness of microbial networks were calculated as described in Yuan et al. (2021). Based on differences in ASV abundance within modules in the co-occurrence network diagram, the within-module connectivity (*Zi*) and among-module connectivity (*Pi*) for each node were calculated, and keystone taxa were identified within the modules (Shi et al., 2016). Nodes were categorized into four distinct types based on their calculated *Zi* and *Pi* values: module hubs (*Zi* > 2.5), network hubs (*Zi* > 2.5 and *Pi* > 0.62), connectors, and peripherals (*Zi* < 2.5 and *Pi* < 0.62) (Zhou et al., 2010).

To elucidate the influence of heavy metals on bacterial community assembly, four ecological models were employed: the normalized stochasticity ratio (NST), Sloan neutral community model (NCM), β-nearest taxon index (βNTI), and infer community assembly mechanisms by phylogenetic-bin-based null model analysis (iCAMP). Each model offers unique insights into the underlying processes shaping microbial communities. NST was used to quantify the balance between stochastic and deterministic processes, with values below 50% indicating deterministic dominance and those above 50% reflecting stochasticity (Ning et al., 2019). The Sloan NCM provided a probabilistic framework to evaluate the role of neutral processes in microbial community assembly, taking into account factors such as dispersal limitation and ecological drift (Sloan et al., 2006). βNTI was applied to assess the phylogenetic turnover between communities, enabling the identification of deterministic processes such as homogeneous or heterogeneous selection (Stegen et al., 2012). Finally, iCAMP integrated phylogenetic and ecological information to dissect community assembly into specific deterministic processes (e.g., homogeneous and heterogeneous selection) and stochastic processes (e.g., dispersal limitation, homogenizing dispersal, and drift) based on βNRI and RC metrics (Ning et al., 2020). Together, these models provided a comprehensive framework to investigate how elemental variations influence bacterial community assembly through deterministic and stochastic processes.

### 2.5 Data availability

All the bacterial raw sequences have been deposited to GenBank Short Read Archive (PRJNA1153714).

## 3 Results

### 3.1 Differences in soil physicochemical properties and enzyme activities among different pollution areas

The contents of elements in soils are shown in Table 1. Soil water content, pH, AK, and AP did not show significant difference among regions (*p* > 0.05). TN, TK, and SOC were significantly higher in selenium-contaminated soils (LSe and HSe) than in Control, while AN was the lowest in HSe soils (*p* < 0.05). Soil K, Mn, Ba, Ti, Co, Ga, and Rb contents were significantly higher in Control soils than in LSe and HSe soils (*p* < 0.05). In addition, the contents of Se, Cr, Ni, Cu, and V were significantly higher in Se-contaminated than in Control soil (*p* < 0.05), with the most significant changes observed in HSe soils, where the increase ranged from 2 to 74 times. However, the contents of other elements, including K, Ce, P, Zn, Na, Mg, Ca, Fe, La, Li, Be, Ti, Cr, Co, Ni, Sr, Mo, Pb, V, Sc, Th, Nb, and As did not show significant difference among regions (*p* > 0.05).

**Table 1:** Soil physiochemical properties in different samples.

Category	Indexes	Control	LSe	HSe
Soil nutrients	Water content (%)	15.28 ± 0.73a	13.8 ± 1.83a	18.97 ± 12.91a
	pH	4.53 ± 0.43a	4.95 ± 0.21a	4.07 ± 0.71a
	AK (mg/kg)	76.1 ± 18.23a	86.2 ± 35.8a	35.03 ± 13.23a
	AP (mg/kg)	0.91 ± 0.2a	35.89 ± 14.87a	54.86 ± 68.65a
	TN (g/kg)	1.36 ± 0.13b	4.54 ± 1.21a	3.02 ± 0.58ab
	TP (g/100 g)	0.04 ± 0b	0.22 ± 0.02ab	1.58 ± 1.05a
	TK (g/100 g)	1.83 ± 0.05b	1.26 ± 0.09a	1.23 ± 0.25a
	SOC (g/kg)	7.53 ± 0.35c	54.97 ± 9.15b	106.71 ± 34.32a
	AN (mg/kg)	122.4 ± 10.61b	186.94 ± 11.76a	44.28 ± 20.67c
Metal elements	Se (mg/kg)	5.75 ± 0.72c	215.28 ± 22.32b	372.52 ± 10.56a
	K (g/kg)	20.62 ± 2.16a	12.15 ± 0.16b	11.63 ± 0.8b
	Ce (mg/kg)	62.96 ± 28a	39.56 ± 7.59a	18.81 ± 11.22a
	P (g/kg)	0.34 ± 0.03a	1.85 ± 0.13a	14.14 ± 10.63a
	Zn (mg/kg)	76.27 ± 8.67a	36.12 ± 5.38b	47.73 ± 17.4ab
	Na (g/kg)	3.51 ± 0.72a	0.97 ± 0.36a	4.01 ± 3.15a
	Mg (g/kg)	15.7 ± 1.28a	13.07 ± 0.8a	5.99 ± 2.28b
	Ca (g/kg)	2.85 ± 0.79a	2.91 ± 0.06a	1.48 ± 0.14b
	Mn (g/kg)	0.63 ± 0.06a	0.21 ± 0.07b	0.05 ± 0.03c
	Fe (g/kg)	52.43 ± 3.35a	25.87 ± 5.51a	31.8 ± 30.12a
	La (mg/kg)	30.45 ± 7.83ab	50.01 ± 5.44a	24.77 ± 12.47b
	Ba (mg/kg)	466.26 ± 66.22a	268.46 ± 40.85b	213.12 ± 19.68b
	Li (mg/kg)	49.43 ± 2.3a	53.35 ± 2.11a	40.64 ± 10.51a
	Be (mg/kg)	1.89 ± 0.05a	2.22 ± 0.22a	1.67 ± 0.53a
	Ti (g/kg)	5.1 ± 0.24a	3.03 ± 0.56b	2.39 ± 0.49b
	Cr (mg/kg)	80.9 ± 4.45b	513.35 ± 68.28a	521.09 ± 75.25a
	Co (mg/kg)	23.18 ± 2.14a	4.13 ± 1.51b	2.25 ± 1.79b
	Ni (mg/kg)	39.62 ± 3.64b	60.81 ± 13.28b	192.03 ± 68a
	Cu (mg/kg)	47.54 ± 1.92c	150.88 ± 2.08a	98.59 ± 8.94b
	Ga (mg/kg)	27.76 ± 1.81a	20.1 ± 2.24b	10.24 ± 3.59c
	Sr (mg/kg)	70.77 ± 9.2a	76.13 ± 10.31a	28.8 ± 6.1b
	Mo (mg/kg)	2.99 ± 0.63a	143.79 ± 17.98a	360.68 ± 372.3a
	Pb (mg/kg)	33.69 ± 1.13a	26.28 ± 1.77a	24.4 ± 10.1a
	V (mg/kg)	116.65 ± 7.09b	1409.08 ± 182.42a	1408.72 ± 835.09a
	Sc (mg/kg)	13.12 ± 3.64a	12.57 ± 2.69a	11.01 ± 3.72a
	Th (mg/kg)	13.22 ± 4.47a	10.32 ± 0.98a	6.76 ± 1.91a
	Rb (mg/kg)	133.84 ± 7.49a	78.91 ± 8.67b	45.81 ± 6.23c
	Nb (mg/kg)	27.6 ± 0.33a	19.54 ± 6.1ab	13.56 ± 4.77b
	As (mg/kg)	10.23 ± 2.55a	4.99 ± 1.35b	8.9 ± 0.43ab

AK, available potassium; AP, available phosphorus; TN, total nitrogen; TP, total phosphorus; TK, total potassium; SOC, soil organic carbon; AN, alkali-hydrolyzed nitrogen. The gray cells indicated the heavy metal elements were significantly higher in HSe group. Data are presented as mean ± standard deviation, with different letters indicating statistically significant differences between treatments at *p* < 0.05.

### 3.2 Effects of different pollution areas on soil bacterial community composition and α-diversity

**Figure 1:**
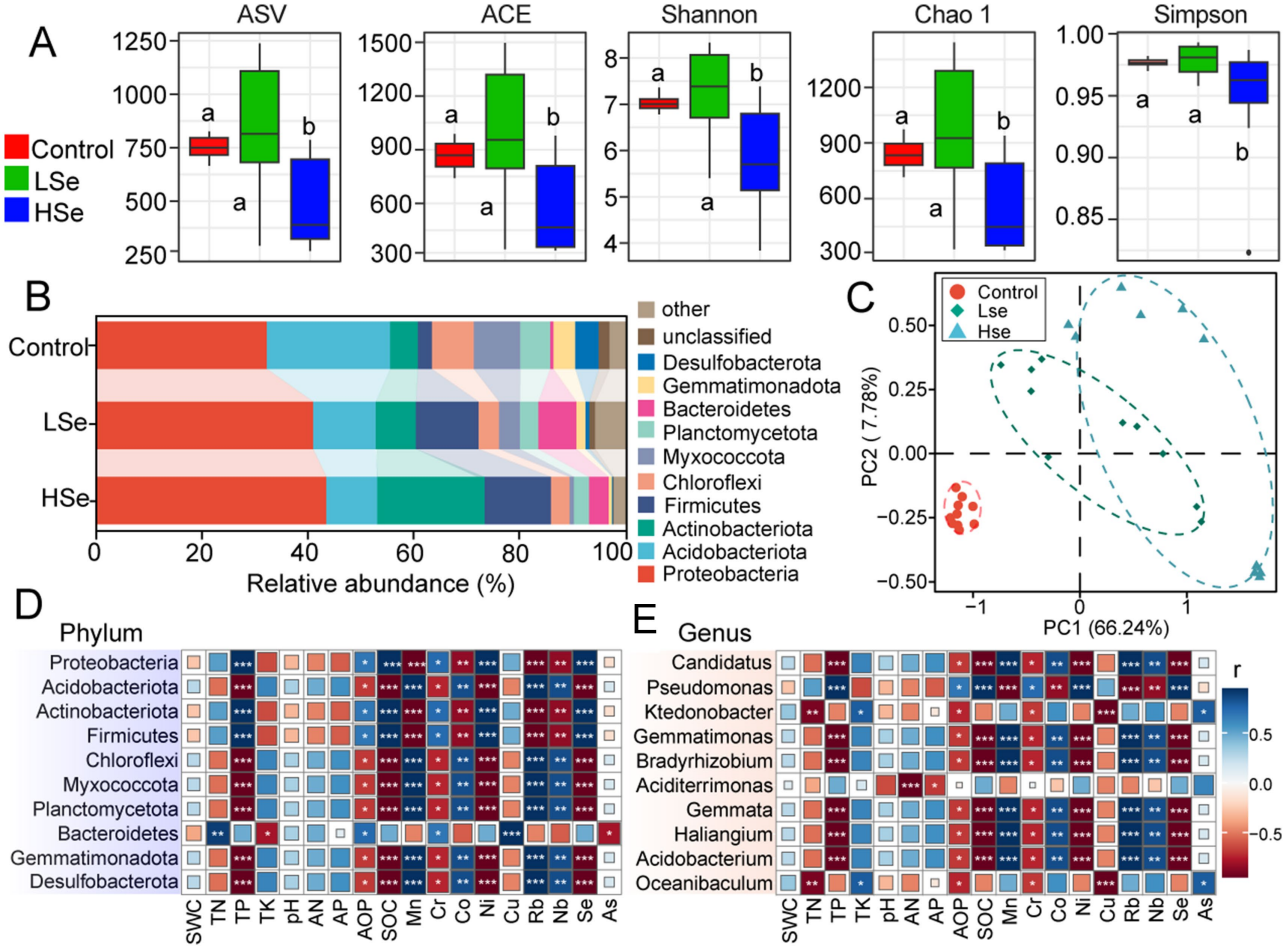
Alpha- and beta-diversity indices of microbial communities in Se-contaminated soils. **(A)** Alpha- diversity indices. **(B)** Relative abundance of microbial phyla across different soil samples. **(C)** Correlation analysis between soil physicochemical properties and relative abundance. Correlation analysis of microbial phyla **(D)** and genus **(E)** with soil physicochemical properties in Se-contaminated soils. ^***^p < 0.001, ^**^p < 0.01, and ^*^p < 0.05.

After filtering out non-target sequences (including chloroplast, mitochondrial, archaeal, and unassigned sequences), a total of 2,105,317 high-quality bacterial sequences were obtained from 30 samples, with an average of 70,177 sequences per sample. As shown in Figure 1A, 5,027 bacterial ASVs were identified across all samples. All alpha diversity indexes, including ACE, Shannon, Chao1 and Simpson were significantly lower in HSe group compared to the LSe and Control groups (*p* < 0.05) (Figure 1A).

The effects of varying Se concentrations and heavy metals on soil microbial communities were primarily reflected in the community composition at the phylum level (Figure 1B). Proteobacteria, Acidobacteriota, Actinobacteriota, and Firmicutes were the four most abundant phyla. In the Control group, Proteobacteria, Actinobacteriota, and Firmicutes exhibited the lowest relative abundances, but their relative abundances increased with higher Se concentrations. Specifically, Proteobacteria increased from 32.05 to 43.30%, Actinobacteriota increased from 5.21 to 20.29%, and Firmicutes from 2.66 to 12.56%. Interestingly, Acidobacteriota showed the opposite trend, decreasing from 23.32 to 9.64%. Apart from these phyla, most other phyla within the top 10 also exhibited a decline in relative abundance. For example, Chloroflexi decreased from 7.95 to 3.51%, Myxococcota from 8.78 to 0.83%, Planctomycetota from 5.65 to 2.84%, Gemmatimonadota from 4.12 to 3.66%, and Thermodesulfobacteriota from 4.44 to 0.17%. The PCA plot demonstrated clear clustering of microbial communities between the Se-contaminated (LSe and HSe) and the Control soils, with the first two principal components explaining 66.24 and 7.78% of the total variance, respectively (Figure 1C). These results indicate distinct microbial community compositions under varying levels of Se and other heavy metal exposure. This was further confirmed by statistical analyses, including ANOSIM (*p* = 0.009), MRPP (*p* = 0.001), and Adonis (*p* = 0.001), all of which demonstrated significant differences between groups (*p* < 0.05) (Table 2).

**Table 2:** Significance tests of the effects of different samples on the bacterial community with three different statistical approaches.

	Adonis		ANOSIM		MRPP	
	F	P	R	P	δ	F
Samples	5.2774	0.001	0.3136	0.001	0.6012	0.001

Correlation analysis revealed variations in the adaptability and ecological functions of microbial communities under specific environmental conditions (Figures 1D,E). At the phylum level (Figure 1D), Proteobacteria showed a significant positive correlation with SOC and Se, while Acidobacteriota exhibited a significant negative correlation with the same variables. Actinobacteriota demonstrated positive correlations with heavy metals such as Mn, Cr, and Cu, indicating potential heavy metal tolerance. In contrast, Gemmatimonadota showed negative correlations with multiple environmental variables, suggesting growth limitations. At the genus level (Figure 1E), *Candidatus* displayed significant negative correlations with AP and Se, while *Pseudomonas* had strong positive correlations with various environmental variables, particularly heavy metals like Cr and Cu. *Ktedonobacter* showed a positive correlation with Se and As, whereas *Gemmatimonas* exhibited negative correlations with AP and SOC, indicating unfavorable conditions for its growth.

### 3.3 Relationship between environmental variables and microbial communities in different pollution areas

**Figure 2:**
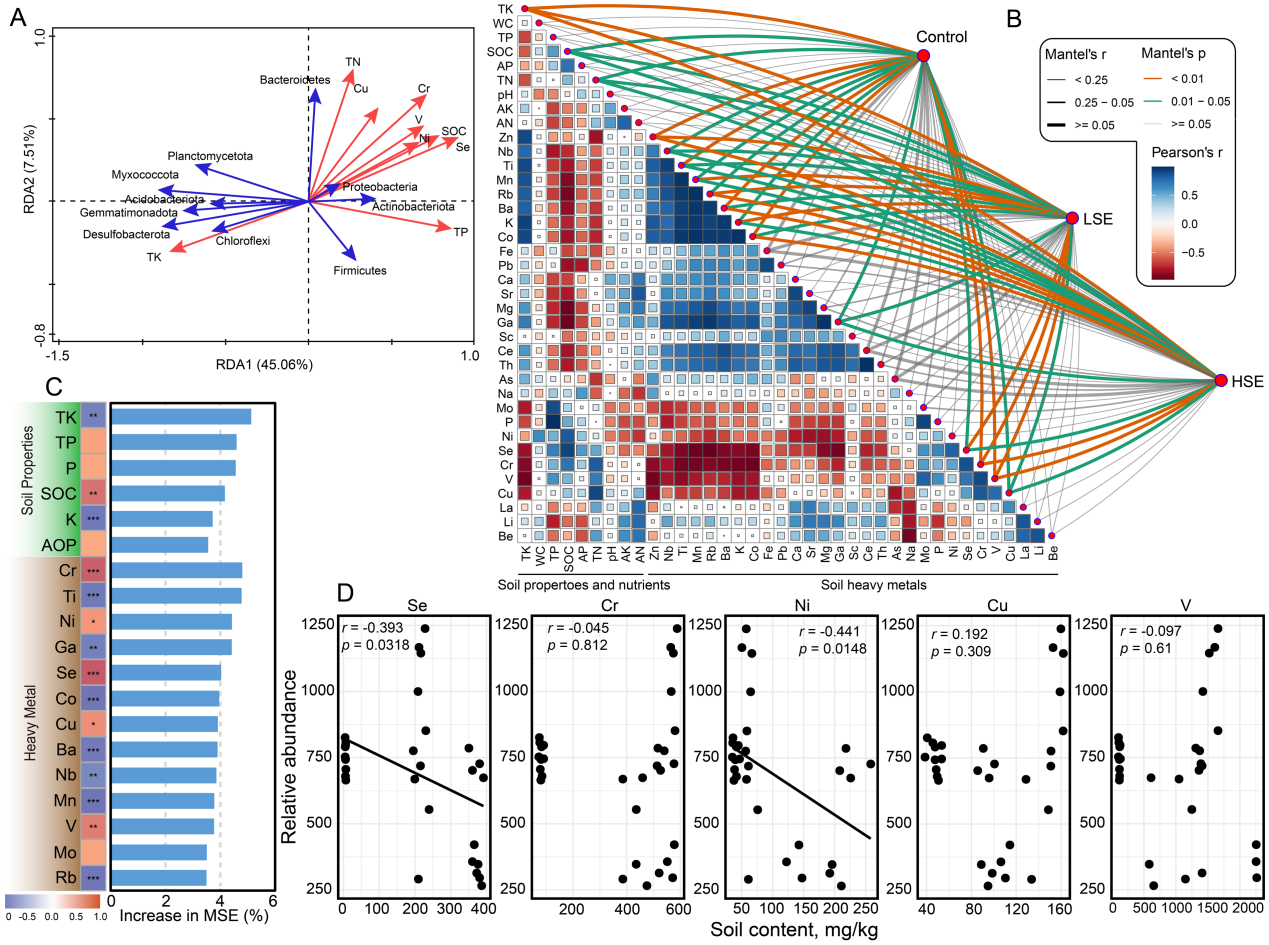
Effect of heavy metals and soil properties on bacterial community diversity. **(A)** Redundancy analysis (RDA) showing the relationships between soil properties, heavy metals, and microbial community compositions. Red arrows represent soil properties and heavy metals, while blue arrows represent microbial taxa. **(B)** Mantel test results visualizing the correlations between soil properties, heavy metals, and microbial communities, with Pearson’s correlation coefficients color-coded. Line thickness indicates the strength of Mantel’s *r*, and significance levels are indicated by line type. **(C)** Random forest analysis ranking the importance of soil properties and heavy metals in influencing microbial community compositions, with the percentage increase in mean squared error (MSE) shown. **(D)** Pearson correlation between soil heavy metal (Se, Cr, Ni, Cu, and V) contents and relative abundance of bacteria. ^**^Asterisks indicate significance levels: ^***^p < 0.05, ^**^p < 0.01, and ^***^p < 0.001.

The microbial community structure was significantly influenced by environmental variables, as revealed through various multivariate analyses. The RDA plot identified the major environmental factors shaping microbial community composition. Soil Se, TN, SOC, and TP were the primary drivers of microbial variation and were correlated with specific microbial taxa (Figure 2A). For instance, Proteobacteria, Actinobacteriota, Firmicutes, and Bacteroidetes showed strong associations with elevated Se and heavy metal concentrations, while other phyla were more closely linked to TK gradients. Mantel test analysis was conducted to explore the potential abiotic factors driving variations in the soil microbial community across different Se-contamination levels. The analysis revealed that TK, SOC, TN, Zn, Nb, Ti, Mn, Rb, Ba, K, Co, Ga, Se, Cr, V, and Cu were significantly and positively correlated with microbial community composition across all groups (Figure 2B). Additionally, heavy metals such as Cr and Cu showed moderate positive correlations with the microbial community, indicating their potential role in shaping community structure. In contrast, certain environmental variables, such as K and Ba, displayed weaker or no significant correlations with microbial community composition. Notably, the strength of these correlations varied between LSe and HSe soils, suggesting that the impact of environmental factors on microbial communities is influenced by the degree of Se contamination. These findings highlight the complex interactions between biotic and abiotic factors in shaping soil microbial communities under Se contamination.

Random forest analysis identified TK as the most critical soil property influencing microbial community structure, with TK showing the highest increase in mean squared error (MSE) (Figure 2C). Multiple heavy metal elements (Cr, Ti, Ni, Ga, and Se) were the most influential factors, further highlighting the combined importance of soil fertility and heavy metal contamination in shaping microbial community dynamics. Pearson correlation analysis demonstrated a significant negative relationship between bacterial relative abundance and soil Se and Ni concentrations (*p* < 0.05) (Figure 2D). At the phylum level, Proteobacteria, Actinobacteriota, and Bacteroidetes exhibited positive correlations with the five heavy metal elements (Supplementary Table S1). Additionally, analysis of soil Se content and the top 20 most abundant genera revealed that *Pseudomonas*, *Staphylococcus*, *Candidatus Solibacter*, and *Serratia* were positively correlated with the majority of these heavy metals (Supplementary Table S1). Together, these analyses underscore the complex interplay between heavy metals and soil properties in shaping microbial community structure.

### 3.4 Microbial co-occurrence networks in different pollution areas

**Figure 3:**
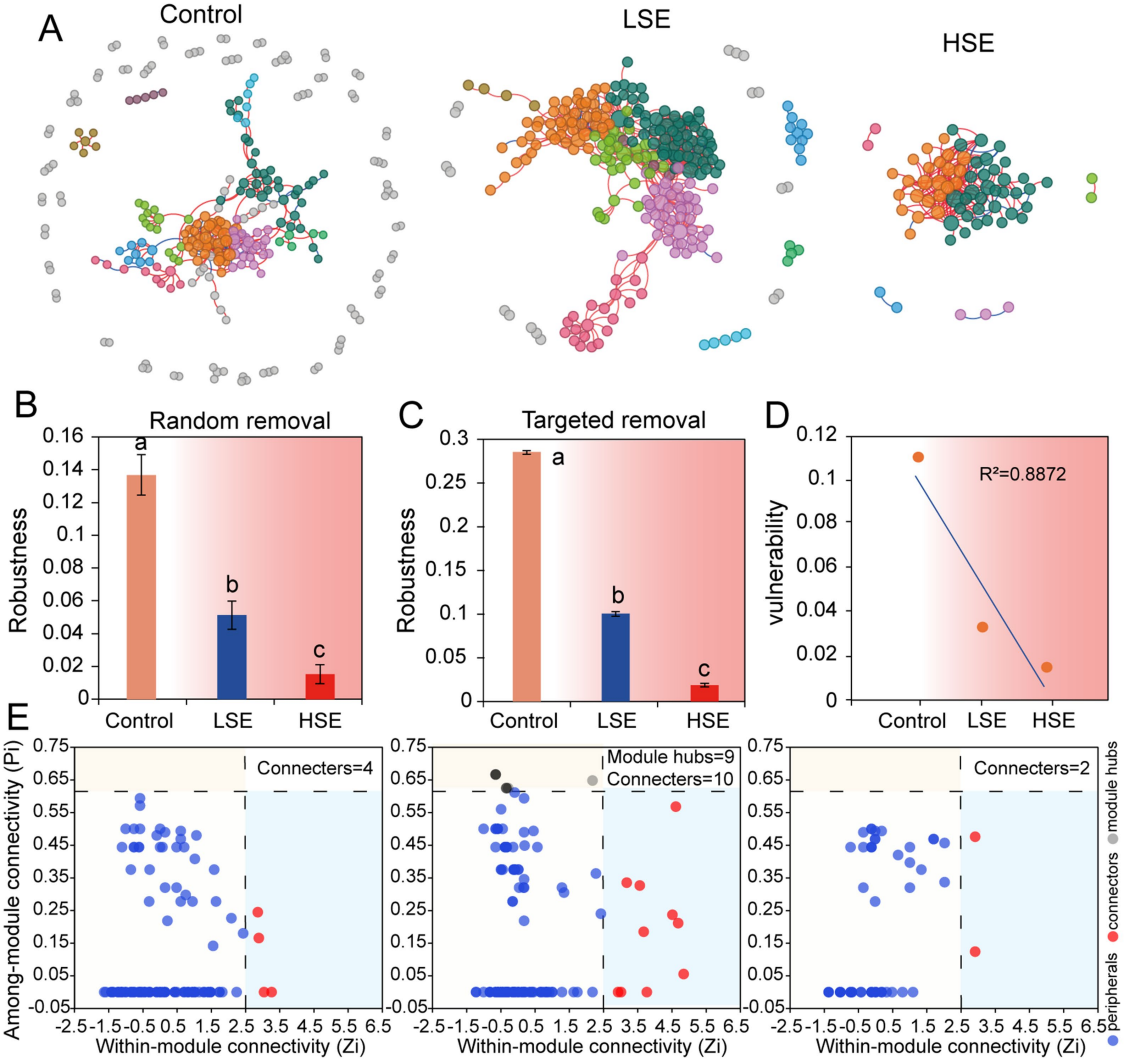
Co-occurrence network analysis of soil microbial community based on Pearson’s correlation analysis. **(A)** Blue and red lines represent significant negative and positive correlations, respectively. The nodes indicate the ASVs, and the color of node represents the model hub. Robustness is calculated as the proportion of remaining species in the community after randomly removing 50% of the nodes **(B)** or targeted hubs **(C)**, while vulnerability **(D)** is determined by the highest node vulnerability in each network. **(E)** Putative keystone taxa in the different networks based on *Pi* and *Zi*. The solid circle represents an ASV.

The bacterial co-occurrence network results demonstrated significant shifts in the microbial co-occurrence network structures under Se-contamination soils. In the Control group, the network exhibited a more modular structure with higher total nodes (257) and links (360), as well as a lower degree centralization (CD = 0.052) and higher average clustering coefficient (avgCC = 0.161) (Table 3), indicating a stable and well-connected network. The presence of keystone ASVs, such as Myxococcota, Proteobacteria, Armatimonadota, and Firmicutes, in the Control group module hubs highlights a diverse and functional microbial community (Figure 3E and Table 4). However, under the LSe group, the microbial network displayed increased total links (551) and average degree (4.322), along with reduced modularity (0.607), suggesting a denser but less modular network with increased connectivity. Keystone taxa in the LSe group predominantly belonged to Proteobacteria and Nitrospirota, which were identified as both connectors and module hubs, indicating their critical role in maintaining microbial interactions and network stability under low Se stress.

**Table 3:** Topological properties of empirical networks of different bacterial communities and their associated random ecological networks.

Network name	Topological properties	Control	LSe	HSe
Empirical	Similarity threshold	0.9	0.94	0.9
	Total nodes	257	255	67
	Total links	360	551	181
	Centralization of degree (CD)	0.052	0.157	0.275
	Average degree (avgk)	2.802	4.322	5.403
	Average path distance (GD)	5.962	4.275	2.845
	Average clustering coefficient (avgCC)	0.161	0.136	0.065
	Centralization betweenness (CB)	0.132	0.21	0.16
	Modularity	0.751	0.607	0.84
Random networks	Modularity	0.626 ± 0.009	0.443 ± 0.006	0.779 ± 0.007
	Average path distance (GD)	4.442 ± 0.099	3.335 ± 0.049	2.662 ± 0.056
	Average clustering coefficient (avgCC)	0.17 ± 0.006	0.088 ± 0.012	0.089 ± 0.016

**Table 4:** Affiliation of keystone ASVs in *Zi-Pi* graph.

Keystone taxa	Nodes	Affiliations (phylum)
Control module hubs	ASV129	Myxococcota
	ASV237	Proteobacteria
	ASV928	Armatimonadota
	ASV1686	Firmicutes
LSe connectors	ASV557	Proteobacteria
	ASV623	Nitrospirota
	ASV916	Proteobacteria
	ASV1634	Proteobacteria
	ASV1636	Proteobacteria
	ASV1863	Proteobacteria
	ASV1876	Proteobacteria
	ASV2095	Proteobacteria
	ASV2818	Proteobacteria
LSe module hubs	ASV58	Proteobacteria
	ASV92	Proteobacteria
	ASV101	Nitrospirota
	ASV132	Proteobacteria
	ASV133	Proteobacteria
	ASV251	Proteobacteria
	ASV261	Proteobacteria
	ASV284	Proteobacteria
	ASV432	Proteobacteria
	ASV702	Proteobacteria
HSe module hubs	ASV24	Acidobacteriota
	ASV105	Acidobacteriota

Module hubs: *Zi* > 2.5, connectors: *Pi* > 0.62.

In contrast, high Se exposure drastically reduced network complexity (Figure 3A), with total nodes decreasing to 67 and links to 181, accompanied by increased degree centralization (CD = 0.275) and a lower clustering coefficient (avgCC = 0.065). The reduced average path distance (2.845) and higher modularity (0.84) in HSe reflect a fragmented and simplified network structure, suggesting a breakdown in microbial interactions and niche differentiation under high Se and other heavy metal stress. Keystone ASVs in the HSe group were primarily from Acidobacteriota, highlighting the resilience of specific taxa in highly stressed environments (Figure 3E and Table 4). Random and targeted node removal analyses further revealed that the robustness of microbial networks decreased significantly with increasing Se and other heavy metal contamination (Figures 3B,C), with HSe networks exhibiting the highest vulnerability (*p* < 0.05) (Figure 3D). Collectively, these findings demonstrate that selenium contamination disrupts microbial co-occurrence patterns, reduces network complexity, and alters keystone taxa affiliation, with high Se and other heavy metal stress leading to significant microbial community fragmentation and functional instability.

### 3.5 Ecological processes governing bacterial community assembly in different pollution areas

**Figure 4:**
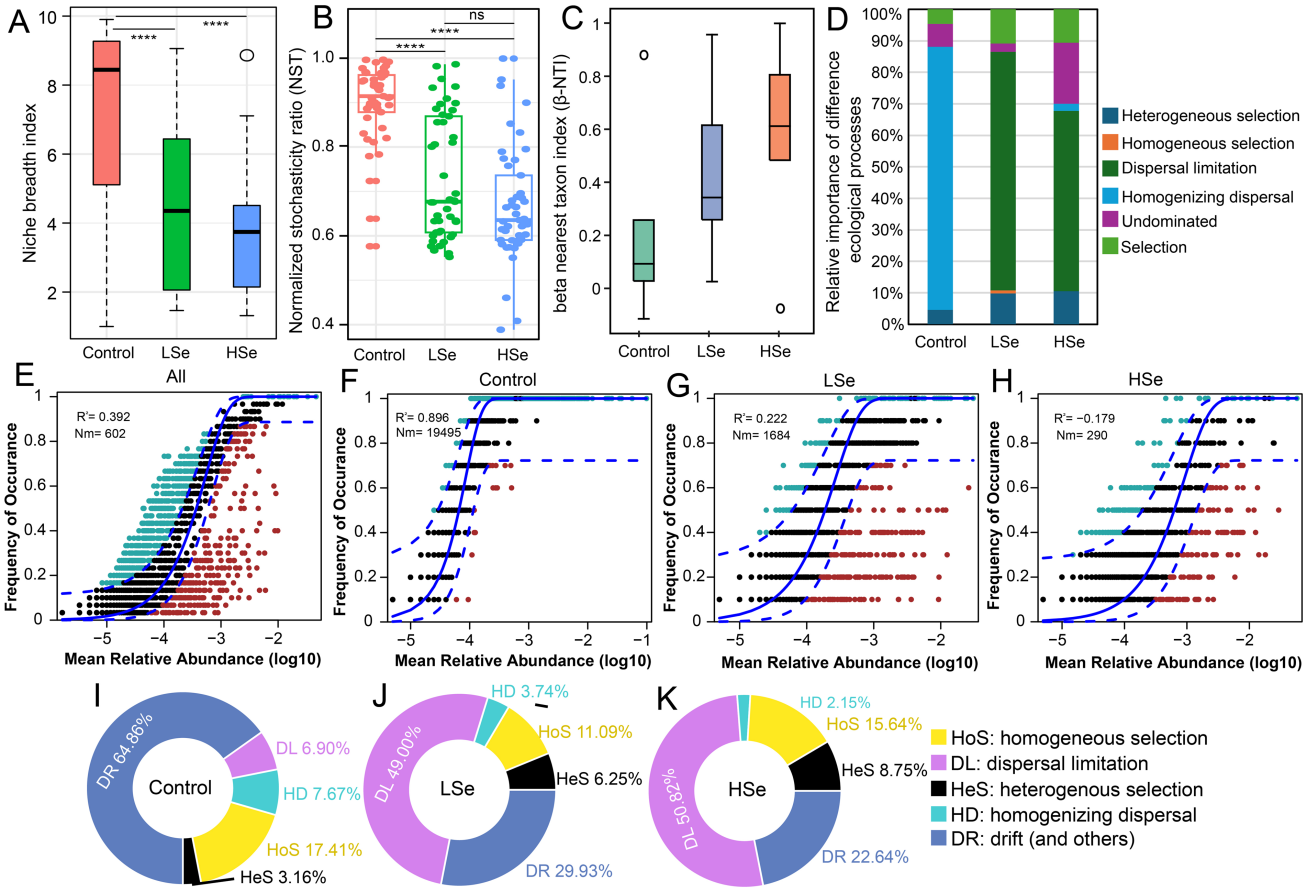
Impact of selenium contamination on bacterial community assembly processes. **(A)** Niche breadth across different samples. **(B)** Normalized stochasticity ratio (NST) based on Jaccard distances. **(C)** Distribution of beta nearest taxon index (β-NTI) among different samples. **(D)** Relative importance of different ecological processes, including heterogeneous selection, homogeneous selection, dispersal limitation, homogenizing dispersal, and drift, highlighting increased deterministic processes (heterogeneous selection). **(E–H)** Sloan neutral community model (NCM) fitting for different soils. **(I–K)** The relative contributions of stochastic and deterministic processes based on the infer community assembly mechanisms by phylogenetic-bin-based null model analysis (iCAMP).

To clarify the influence of heavy metals on bacterial community assembly, four ecological models were employed to dissect the relative roles of deterministic and stochastic processes. First, niche breadth and the normalized stochasticity ratio (NST) were analyzed to assess community adaptability and the balance between stochasticity and determinism. The Control group displayed the broadest niche breadth, reflecting high adaptability to environmental conditions (Figure 4A). However, with increasing Se and other heavy metal concentrations, especially in the HSe group, niche breadth significantly decreased (*p* < 0.05). This decline indicates that elevated stress reduced species adaptability, leading to a drop in microbial diversity and a shift in the dominance of stochastic and deterministic processes. NST values calculated using Jaccard, Bray–Curtis, and weighted UniFrac metrics revealed that stochastic processes controlled the majority of bacterial community assembly in all groups (89.87, 73.10, and 66.79% for Control, LSe, and HSe, respectively) (Figure 4B). While stochastic processes prevailed in the Control and LSe groups, a notable decline in NST in the HSe group suggested a growing contribution of deterministic processes. This pattern likely results from increased environmental pressure, which favors the survival of specific taxa, causing a shift toward more deterministic community assembly.

To better identify the ecological drivers, βNTI values were used to assess community assembly mechanisms. βNTI values between −2 and 2 indicated that stochastic processes predominated across all samples (Figure 4C). However, a closer analysis revealed that in the Control group, bacterial communities were primarily regulated by homogenizing dispersal, while in the LSe and HSe groups, dispersal limitation became the key stochastic factor influencing community assembly (Figure 4D). To further investigate the role of stochastic processes, we applied the Sloan neutral community model (NCM). The *R*^2^ values of 0.896, 0.222, and −0.179 for the Control, LSe, and HSe groups (Figures 4E–H) highlighted that stochasticity significantly influenced bacterial community assembly in the absence of Se and other heavy metal stress but decreased with Se contamination. The *m* value, reflecting microbial dispersal ability, was substantially higher in the Control group (0.971) compared to Se-polluted soils (0.014–0.084) (Supplementary Table S2), suggesting that Se and other heavy metal contamination severely constrains microbial dispersal.

iCAMP analysis provided additional insights into the contributions of deterministic and stochastic processes (Figures 4I–K). In the Control group, stochastic processes (sum of DR, DL, and HD), including drift and homogenizing dispersal, explained the majority of bacterial community variation (72.53%), whereas deterministic processes accounted for 27.47% (Figure 4I). In contrast, stochastic processes dominated in the HSe group, explaining 76.21% of community variation, while deterministic processes contributed only 24.79% (Figure 4K). This shift highlights the increasing impact of selection pressure and dispersal constraints under Se pollution, as also supported by βNTI results. Notably, drift contributions declined significantly as heavy metal content increased (from 64.86 to 22.64%), while dispersal limitation rose sharply (from 6.9 to 50.82%). Overall, dispersal limitation (49–50.82%) emerged as the most significant stochastic factor driving bacterial community assembly under Se and other heavy metal stress, followed by drift (22.64–29.93%).

## 4 Discussion

### 4.1 Interactions between selenium, other heavy metals, and soil properties

In previous studies, conflicting results have emerged regarding the relationship between soil Se content and other elements such as Cu, Ni, Cd, Pb, and As. For instance, Jiang (2011) reported a negative correlation between Se and these elements, while Guo (2011) observed a positive correlation, findings that were further supported by Li et al. (2023). In our study, Se showed a negative correlation with As and Pb but a positive correlation with Cu and Ni. These differing correlations can be attributed to several factors, including the chemical properties of Se and the presence of other contaminants in the soil. Se exists in different forms (e.g., selenate, selenite, and elemental Se), each of which interacts differently with other elements depending on its concentration and speciation (Fernández-Martínez and Charlet, 2009). Additionally, other heavy metals or organic matter can influence how Se interacts with these elements, affecting the overall correlation patterns (Xiao, 2014). Plant species may also play a role in shaping these correlations. Certain plants, particularly Se-hyperaccumulator plants (*Cardamine enshiensis*), can alter the mobility and bioavailability of Se and other heavy metals through processes such as ion exchange, root exudation, and metal uptake (Kumar et al., 2017; Li et al., 2018). These plants, known for their ability to absorb high concentrations of metals, can influence the solubility of Se and its interactions with other metals like Cu, Ni, and Pb (El Mehdawi and Pilon-Smits, 2012).

Another significant issue associated with Se is its potential to exacerbate heavy metal pollution in soil. The observed coexistence relationships between Se and Cr, Ni, and Cu in Se-rich soils suggest that Se may enhance the mobility or bioavailability of these metals. For instance, selenate ions can form stable complexes with metal ions, increasing their solubility and transport in soil (Lenz and Lens, 2009; Torres et al., 2010). This effect is particularly relevant in soils with high Se content, where heavy metals may exceed regulatory thresholds due to increased mobilization.

Microbial activity also plays a critical role in modulating the effects of heavy metals on soil properties. Microbes can reduce selenate and selenite to elemental Se or volatilize it as dimethyl selenide, which can influence the mobility and bioavailability of Se (Jiang et al., 2024). Furthermore, the influence of Se on microbial and plant communities can indirectly affect heavy metal dynamics. For example, appropriate Se supplementation has been shown to reduce heavy metal accumulation in crops by modulating plant uptake pathways (Zhu et al., 2022). Additionally, microbial activity can affect soil pH and redox potential, which in turn influences the solubility of metal ions (Husson, 2013). This complex interplay between microbial activity, metal contamination, and soil properties underscores the difficulty in predicting the specific impact of Se and other heavy metals on soil physicochemical characteristics.

### 4.2 Effects of Se and other heavy metals on soil microbial communities

Heavy metal contamination significantly affects soil microbial communities due to its persistence and slow degradation in the environment. Se, as both an essential trace element and a potential contaminant, exerts a more complex effect on soil bacterial communities and their diversity (Kushwaha et al., 2022). At low concentrations, Se can promote microbial activity by participating in microbial metabolism (Jiang et al., 2024). However, at high concentrations, its toxicity inhibits microbial growth, disrupts cellular processes, and damages enzyme functions, leading to a decline in microbial diversity (Kushwaha et al., 2022). In this study, Se and other heavy metal concentration was closely associated with changes in bacterial community composition, with significant shifts observed in the relative abundance of dominant bacterial phyla and genera (Figures 1, 2). Certain selenium-tolerant bacteria, such as Proteobacteria and Actinobacteriota, increased in abundance in Se-contaminated soils, possibly due to their ability to reduce selenates (SeO₄^2−^) or selenites (SeO₃^2−^) to less toxic elemental Se (Se^0^) or volatile Se compounds (Shi et al., 2021). These microbes may play a key role in the biogeochemical cycling of Se. Researchers also found that Se was positively correlated with the growth of nitrogen-fixing bacteria, such as Rhizobiaceae and Frankia, and nitrifying bacteria like Proteobacteria, Firmicutes, and Chloroflexi (Lei et al., 2022; Tang et al., 2024), while Se inhibits the growth of Ascomycota (Yang X. et al., 2022). Moreover, the interaction between Se and other soil properties and pollutants plays an important role in regulating microbial communities. For example, Se can form complexes with other heavy metals (such as Cu and Ni), altering their bioavailability and potentially enhancing their toxicity to microbes or plants (Feng et al., 2021). This phenomenon may explain the observed changes in microbial community composition in soils co-contaminated with Se and heavy metals. Additionally, selenium’s regulation of soil redox potential and pH may indirectly influence microbial communities by altering nutrient and pollutant availability (Kushwaha et al., 2022).

### 4.3 Microbial co-occurrence network structure in response to Se and other heavy metal contamination

In nature, microbes do not exist in isolation but rather interact and adapt together to environmental pressures (Chun et al., 2021). Soil microbial networks can reveal these interactions and reflect the community’s response to environmental changes (Zhou et al., 2010). In this study, the microbial network exhibited higher connectivity and lower modularity under low Se and other heavy metal contamination conditions, suggesting denser interactions and less differentiation between microbial groups (Lurgi et al., 2019). This may be due to the selective pressure of low Se and other heavy metal concentrations, which promotes broader interactions among microbial communities (Sun et al., 2022). This phenomenon indicates that low Se and other heavy metal contamination may enhance the synergistic effects between different microbial populations, thus aiding community adaptation and stability. In contrast, the complexity of the microbial network drastically decreased under high Se and other heavy metal exposure. This indicates an increase in microbial interactions or niche-sharing within microbial community due to its smaller, more tightly connected structure (Sun et al., 2022). The toxic effects of high Se and other heavy metal concentrations likely imposed selective pressure, allowing only Se-tolerant species to thrive (Colpaert et al., 2011; Chun et al., 2021). These resilient species became the “hubs” of the network, but their dominance led to reduced diversity and ecological function within the community, causing fragmentation and loss of network functionality (Chun et al., 2021). This change highlights how high Se and other heavy metal concentrations disrupt microbial community dynamics, pushing certain taxa to the core while inhibiting others from growing.

Interestingly, despite the significant reduction in network complexity under high Se and other heavy metal concentrations, microbial community stability and adaptability were enhanced under low Se and other heavy metal contamination. A potential explanation for these observations is that low Se and other heavy metal contamination may promote cooperation and nutrient interactions among microbial communities, while high Se and other heavy metal contamination may limit microbial diversity and interactions, leading to a decline in community stability and resilience (Zaefarian et al., 2011). However, it is important to note that species associations based on correlation analysis may not fully reflect the true interactions among microbes, and some of the negative/positive correlations may be incidental or influenced by environmental factors. Therefore, caution is needed when interpreting these results in microbial community network analysis (Jiao et al., 2021). Nevertheless, negative/positive correlation information remains crucial for identifying potential species relationships within microbial communities, especially in complex environmental conditions, and provides valuable insights for understanding microbial interaction patterns (Jin et al., 2022).

### 4.4 Effects of Se and other heavy metals on microbial community assembly

Se and heavy metal contamination significantly altered the balance between stochastic and deterministic processes in microbial community assembly. This shift is likely due to the significant disturbance of the soil microenvironment caused by high Se and other heavy metal concentrations, such as enhanced toxicity and altered resource allocation, leading to selective enrichment of specific functional groups and exclusion of sensitive ones (Guo et al., 2023). Under low Se and other heavy metal contamination, stochastic processes remained dominant, with homogenizing dispersal promoting community stability (Howeth and Leibold, 2010). This may offer opportunities for microbial coexistence under environmental stress. In contrast, under high Se and other heavy metal contamination, environmental selection effects were clearly intensified, characterized by narrowed ecological niches, limited dispersal capacity, and dominant deterministic processes. This pattern is closely linked to the inhibitory effects of Se and other heavy metal toxicity on microbial diversity and biomass. Additionally, the enhancement of dispersal limitation, as a major stochastic process, likely reflects local adaptation of microorganisms in high-pollution environments (Dai et al., 2023). Tolerant groups, such as Proteobacteria and Actinobacteriota, were preferentially enriched, likely due to their ability to reduce selenate and selenite (Kang et al., 2024), gaining an advantage in niche competition (Jing et al., 2024). These results reveal the complex role of Se and other heavy metal contamination in microbial community dynamics: on one hand, deterministic processes driven by selective pressure enhance the regulation of community structure, while on the other hand, dispersal limitation (as the dominant stochastic process) significantly affects community stability and resilience. Under high Se and other heavy metal contamination, increased environmental stress favors the survival of specific microbial groups, while the influence of random drift on the community is reduced. Moreover, dispersal limitation becomes a key factor in shaping community assembly, especially in high Se and other heavy metal environments, suggesting that microbial distribution is constrained, which may impact the recovery capacity and ecological functions of microbial communities. Such changes in assembly mechanisms may further affect the functional stability and nutrient cycling efficiency of soil ecosystems, which is particularly significant in agricultural soils and pollution remediation scenarios.

## 5 Conclusion

In summary, selenium and heavy metal contamination exerts complex and significant effects on soil microbial communities, influencing both community structure and function. Our findings highlight the dominance of stochastic processes in microbial community assembly, especially in less contaminated environments, while deterministic processes play a more prominent role in selenium-contaminated soils due to heavy metal toxicity. These insights enrich our understanding of the ecological consequences of selenium and heavy metal pollution and provide a theoretical foundation for developing microbiological-based soil remediation strategies aimed at restoring ecosystem balance and functionality.

## Data Availability

The datasets presented in this study can be found in online repositories. The names of the repository/repositories and accession number(s) can be found in the article/Supplementary material.
