# Supplementary material for: Microbial diversity and community assembly in heavy metal-contaminated soils: insights from selenium-impacted mining areas
Source: Front Microbiol. 2025 Apr 14;16:1561678. doi: 10.3389/fmicb.2025.1561678 (PMC12034704; doi:10.3389/fmicb.2025.1561678)
Supplement: Supplementary file 2 [file Table_2.DOCX]

Table S2 The neutral community model (NCM) in different bacterial communities

|  | R^2^ | *N* | *m* | *Nm* |
| --- | --- | --- | --- | --- |
| All | 0.392 | 20085 | 0.030 | 602 |
| Control | 0.896 | 20085 | 0.971 | 19495 |
| LSe | 0.222 | 20085 | 0.084 | 1684 |
| HSe | -0.179 | 20085 | 0.014 | 290 |
